# The Genotoxic and Cytotoxic Effects of Bisphenol-A (BPA) in MCF-7 Cell Line and Amniocytes

**Published:** 2016

**Authors:** Seyed Mohsen Aghajanpour-Mir, Ebrahim Zabihi, Haleh Akhavan-Niaki, Elahe Keyhani, Iman Bagherizadeh, Sajjad Biglari, Farkhondeh Behjati

**Affiliations:** 1 *Cellular & Molecular Biology Research Center, Health Institute, Babol University of Medical Sciences, Babol, Iran.*; 2 *Genetics Research Center, University of Social Welfare and Rehabilitation Sciences, Tehran, Iran.*; 3 *Department of Medical Genetics and Sarem Cell Research Center (SCRC), Sarem Women’s Hospital, Tehran, Iran.*

**Keywords:** Bisphenol-A (BPA), estrogen receptor, MCF-7, amniocyte, chromosome abnormality, classic cytogenetics

## Abstract

Bisphenol-A (BPA) is an industrial xenoestrogen used widely in our living environment. Recently, several studies suggested that BPA has destructive effects on DNA and chromosomes in normal body cells via estrogen receptors (ER). Therefore, BPA could be considered as an important mediator in many diseases such as cancer. However, there are still many controversial issues which need clarification. In this study, we investigated the BPA-induced chromosomal damages in MCF-7 cell line, ER-positive and negative amniocyte cells. Cytotoxicity and genotoxicity effects of BPA were also compared between these three cell groups. Expression of estrogen receptors was determined using immunocytochemistry technique. The cell cytotoxicity of BPA was measured by MTT assay. Classic cytogenetic technique was carried out for the investigation of chromosome damage. BPA, in addition to cytotoxicity, had remarkable genotoxicity at concentrations close to the traceable levels in tissues or biological fluids. Although some differences were observed in the amount of damages between ER-positive and negative fetal cells, interestingly, these differences were not significant. The present study showed that BPA could lead to chromosomal aberrations in both ER-dependent and independent pathways at some concentrations or in cell types yet not reported. Also, BPA could probably be considered as a facilitator for some predisposed cells to be cancerous by raising the chromosome instability levels. Finally, estrogen receptor seems to have a different role in cytotoxicity and genotoxicity effects.

Bisphenol-A (BPA) is an industrial xeno-estrogene which is widely used in the production of polycarbonate plastics, drink containers, baby bottles, epoxy resin lining of food containers, medical devices and dental sealants. BPA is an organic colorless solid compound, with 8 billion pounds yearly production and one hundred tones releasing in atmosphere in 2010 which is increased to 15 billion pounds yearly production and probably more than 200 tones releasing in atmosphere per year during recent years (1-2). BPA has also been detected in a variety of environmental samples, including water, dust, sewage, indoor and outdoor air samples (3). In the last decade, several studies investigated the hazardous effects of BPA which has probably been associated with diabetes, cardiovascular disease, neurobehavioral disorders, recurrent miscarriages, abnormal karyotypes, poly-cystic ovarian syndrome, reproductive impair-ments and cancer (4-14). In 1960, the first chromosomal abnormality associated with cancer was reported using cytogenetics techniques in patients with chronic myeloid leukemia (15). Genomic instability and chromosomal abnorma-lities are well-known common features of cancer (16-17). 

Recent studies have strongly suggested that DNA damage induced by xenoestrogens and estrogen is dependent on estrogen receptors (ERs) (3, 18-19). An in vitro study has indicated the effect of estradiol on radiation-induced chromosome aberrations in human peripheral lymphocytes (20). BPA is considered as an estrogenic endocrine disrupting chemical which exhibits estrogen-like activity (21). BPA binds to ERs that could promote breast cancer (22). 

To date many studies have indicated controversial issues concerning chromosomal aberrations induced by BPA. Although some studies suggested that BPA cannot have a genotoxic effect, some others suggested that BPA exposure can lead to chromosomal abnormalities such as aneuploidy through disruption of meiotic process (23-24) and also genomic structural aberrations like DNA breakage (25). Recent studies have demonstrated that BPA impairs the double-strand break repair machinery in the germline and causes chromosome abnormalities (26). Furthermore, BPA induces synaptic defects, such as end-to-end chromosome associations and asynapsis (27). Nevertheless, it seems that there are no strong evidences to favor BPA as a genotoxic agent in low concentrations which are probably traceable in human biologic fluids or tissues. 

Investigating direct genotoxic effects of BPA on chromosomes, necessitates the exclusion of secondary genotoxic effects of BPA which may occur subsequent to its cytotoxic effects such as apoptosis or necrosis. For this purpose, we used classic cytogenetics method. As cells affected by high cytotoxicity could not be prepared to enter the metaphase stage, then, these kinds of cells will automatically be removed from genotoxicity evaluation of BPA.

To date, different results have been obtained from the study of BPA toxic effects on different cell groups (28). In the present study, we selected MCF-7 cell line which seems to be a suitable representative cell line from breast as one of the main target tissues of BPA. MCF-7 is an ER positive cancerous epithelial cell, with immortal features and high proliferation potential like other cancerous cells (29). These features as well as its high endurance potential to the toxic agents make this cell line a good monitoring system to detect chromosomal aberrations.

The effects of environmental pollutants on fetuses are an important health issue and amniocytes seem to be accessible and suitable representative of fetal cells. Amniocytes are less differentiated cells with higher proliferation potential compared to differentiated cells. 

For the investigation of BPA effects on normal cell population, we have selected ER negative and positive amniocytes, derived from human male and female fetal amnion cells, respectively (30). To the best of our knowledge, there is no other similar study on amniocytes.

## Materials and methods

**Human amniocytes and MCF-7 cell culture**

 MCF-7 cell line was obtained from Pasteur institute, Tehran, Iran. These cells were cultured in RPMI-1640 medium (PAA, Austria) supplemented with 10% fetal bovine serum and 1% antibiotics (penicillin/streptomycin) (Invitrogen, USA) in a humidified atmosphere containing 5% CO2 at 37 °C. Cytogenetically normal human amniocytes were obtained from discardable cell cultures belonging to individuals referred to Sarem Hospital (Tehran, Iran), for some prenatal diagnosis tests. The names and specifications of the subjects were not available for the authors. This project has been approved by the ethical committee of University of Social welfare and Rehabilitation Sciences.

**Cell culture on cover slip and immunocytoche-mistry (ICC)**

Immunocytochemistry (ICC) was performed in order to check the expression of estrogen receptors in amniocytes. MCF-7 cells were used as an ER-positive control sample. 

For performing ICC staining, about 3×104 male and female amniocytes and also MCF-7 were cultured on sterile coverslips. Cells were then fixed and permeabilized with acetone and incubated with mouse anti human estrogen receptor primary antibody (Clone 1D5, Dako, Denmark) for 1 h at room temperature. After that, cells were washed three times with PBS and then incubated with secondary antibodies which was conjugated with Horse Radish Peroxidase (HRP) (Real envision Dako, Denmark), for 30 min at room temperature. Coverslips were washed and covered with chromogen and 3,3'-Diaminobenzidine (DAB) solution (DAKO, Denmark). Hematoxylin was used for counterstaining followed by alcohol and xylene for dehydration. Finally, the coverslips were mounted on slides using mounting media.

**Cell cytotoxicity evaluation using MTT assay**

MTT (3-(4,5-dimethylthiazol-2-yl)-2,5-diphe-nyltetrazolium bromide) assay was done in order to determine the half maximal inhibitory concentration (IC50) of BPA in each cell type and hence the determination of suitable BPA concentrations for the exposure of cell cultures. MCF-7 and amniocyte cells were seeded in complete medium in a 96-well plate at a density of 8x103 and 1x104 cells per well, respectively. After 24 h incubation in 5% CO2 at 37 °C, culture media were replaced by new media containing different concentrations of BPA (0 or control, 0.4, 1, 4, 40, 100 and 400 μg/ml) and cells were incubated for 48 h. After this time period, 100 μl of 5 mg/ml concentrated MTT or tetrazolium salt was added to each well and then the plates were incubated in 5% CO2 at 37 oC for 4 h. Acidic isopropanol was used for dissolving the blue crystals derived from yellow MTT in live mitochondria which lead to a color in each well. The spectrophotometric absorbance of the samples was measured using the micro titer plate (ELISA) reader at the wavelength of 550 nm.

**Preparation of metaphase chromosomes**

Six different concentrations of BPA (0 or control, 0.4, 1, 4, 40, and 100 μg/ml equivalent to 0, 1.75, 4.37, 17.5, 175, 437 μmol, respectively) were used in this study. Acetonitrile was used as the solvent. The final concentration of acetonitrile in cell cultures was 0.6%. 

Cells were seeded in 25 cm3 flasks at an initial density of 1.5x104 cells. RPMI 1640 with 10% fetal bovine serum (FBS) and 1% penicillin/streptomycin was used for MCF-7 cell culture. Amniomax media containing 10% fetal bovine serum (FBS) and 1% penicillin/strep-tomycin was used for the amniocytes. After the cells reached 50-60% confluency, the old medium was replaced with the new one containing different concentrations of BPA and was incubated for further 48 h. Cells were arrested at metaphase stage by adding 50 μl colcemid (Gibco, USA) for 2 h prior to the harvest. Cells were detached with 0.025% trypsin-EDTA (Sigma, Germany). The cell suspension was centrifuged at 1200 X g for 10 min and the pellet was resuspended in 10 ml of hypotonic potassium chloride and incubated for 15 min at 37 oC followed by centrifugation at 1200 X g for 10 min. The plate was resuspended in fixative solution of 3:1 mixture of methyl alcohol and glacial acetic acid. The fixed cells were dropped onto a clean glass slide and then were aged for 24 h at 72 °C. 

**Solid staining and G-banding**

Solid staining has been performed as a standard method for investigation of chromosome and chromatid gaps and breaks. To this end, slides were covered with 10% giemsa stain for 5 min. For GTG banding, slides were rinsed in the pancreatin enzyme solution (Sigma-Aldrich, USA) followed by giemsa stain for 5 min. Then, all the slides were dried and sealed with Entellan mounting medium and were covered by coverslips.

## Results

**Confirmation of the existence of estrogen receptors in MCF-7 and female amniocytes**

**Fig. 1:**
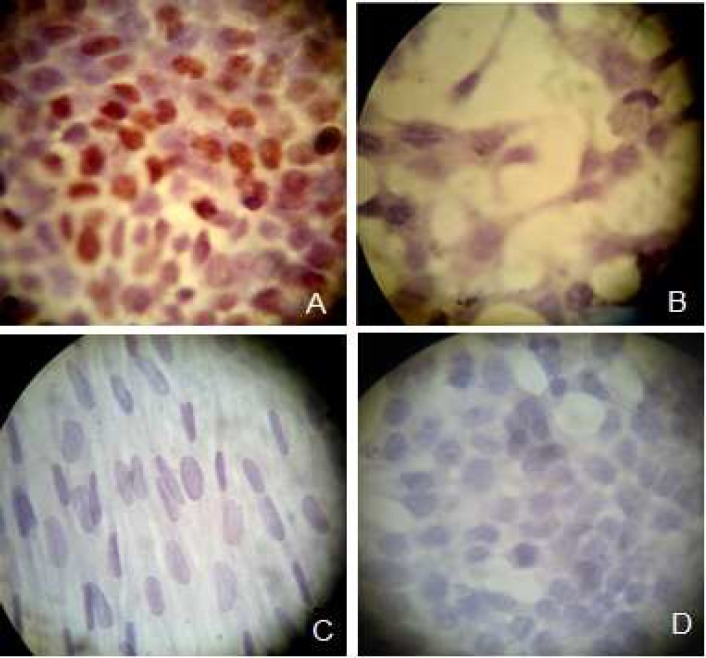
Immunocytochemistry staining of the estrogen receptors in the cells. MCF-7 as the positive control (A), female amniocytes (B), male amniocytes (C) and MCF-7 cells without the estrogen receptor primary antibodies as the negative control (D

In order to evaluate the presence of estrogen receptors, ICC was used for all three cell groups. For this purpose, MCF-7 was used as a positive control which clearly expressed ERs (Figure 1- A). As it was expected, results showed that unlike the male amniocytes, the females expressed ER (Figure 1- B, C). To evaluate the specificity of this method we used MCF-7 without anti estrogen receptor as a negative control (Figure 1- D).

**Cell cytotoxicity evaluation of BPA**

**Fig. 2:**
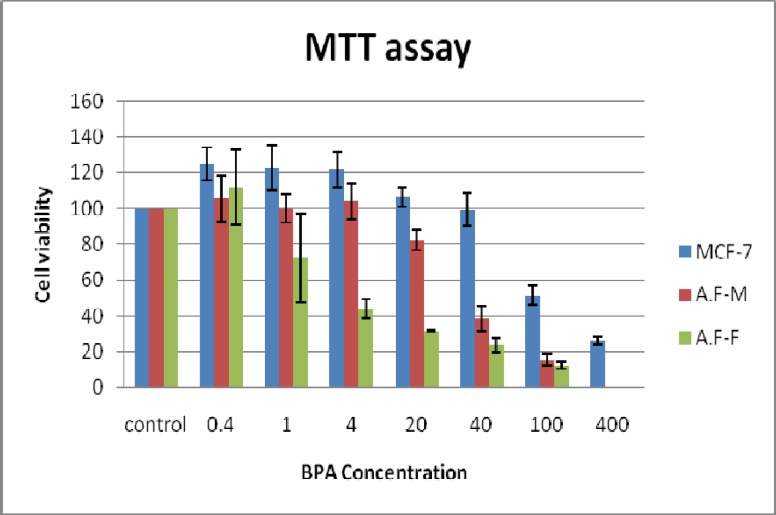
Cytotoxicity evaluation of BPA on MCF-7 cell line using MTT assay, male (A.F-M) and female (A.F-F) amniocytes. The IC50 for MCF-7, male and female amniocytes were about 100, 40 and 4 μg/ml BPA, respectively

For all three cell groups, the related IC50 have been determined. Results of MTT showed that the half maximal inhibitory concentration for MCF-7, male and female amniocytes were about 100, 40 and 4 μg/ml, respectively (Figure 2).

The obtained results suggest that cytotoxicity effects of BPA are dose dependent and different cells with different ER pattern showed completely different susceptibility to BPA. To the best of our knowledge, there was not any similar study on the amniocytes. To determine the minimal genotoxic concentrations of BPA in in vitro, it was decided to use the IC50 concentrations (4, 40 and 100 μg/ml) for all three cell groups as well as 0.4 and 1 μg/ml BPA which also was suggested by other studies (28).

**Induction of chromosomal aberrations in the cells exposed to BPA**

Assessments of the chromosomal abnormali-ties in terms of structural and numerical aberrations have been done to investigate the effects of BPA on three cell types with relatively high proliferation potential that make them more sensitive to the toxic agents.

For MCF-7 which is a cancer cell line with many chromosomal abnormalities, we assessed the abnormalities in the untreated or control group of MCF-7 cells and then compensated it with the other concentrations to exclude it's in born abnormalities. Only structural chromosome abnormalities inclu-ding fragments, chromosome gaps, chromosome breaks, chromatid gaps, chromatid breaks, and chromosome rearrangements such as triradials were scored. The numerical abnormalities were not scored as there was a wide range of chromosome aneuploidies in cells obtained from the MCF-7 cell line. Thus, just the tendency to decrease or increase the chromosomal numbers was considered.

**Structural aberrations**

**Fig. 3:**
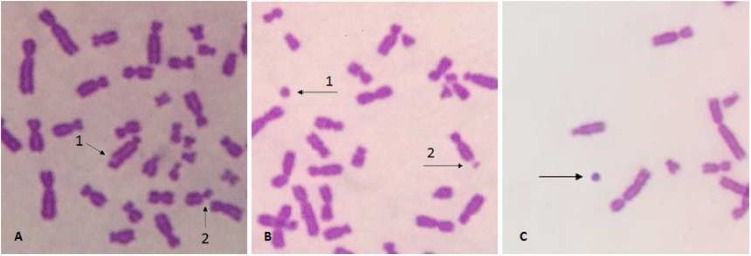
Some structural aberrations. A1&2: chromatid gap and chromatid break, respectively in MCF-7; B1&2: fragment and chromosome gap, respectively in MCF-7; C: fragment in female amniocyte

The complete data of chromosomal structural aberrations are provided in table 1. Some of chromosomal structural aberrations are shown in figure 3. 

**Fig. 4:**
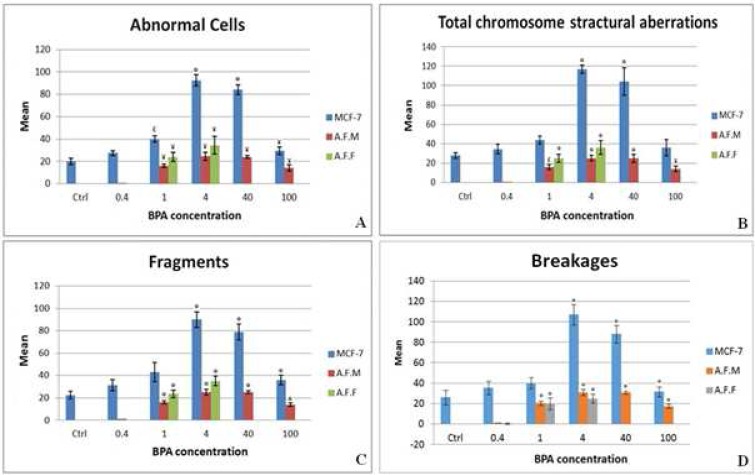
Average amounts of abnormal cells. A: cells with at least one chromosomal structural aberration; B: total structural abnormalities; C,D: two of main structural abnormalities in different concentrations of BPA (μg/ml). AFM: male amniocytes, AFF: female amniocytes,  *: p< 0.001, ¥: p< 0.005, £: p< 0.05

A significant increase of abnormal cells (cells with at least one structural aberration) was observed at 1 μg/ml of BPA for all three cell groups. Althou-gh in MCF-7 cells, a notable increase of abnormal cells (19% increase compared to normal control group) was obvious at the lowest concentration, but it was non-significant (figure 4A).

As it is clear in figure 4B, an unexpected increase of structural aberrations was seen in amniocytes at a dose of 1 μg/ml but not at 0.4 μg/ml of BPA. For MCF-7 cell line, total structural aberrations increased from 0.4 μg/ml, but it was not significant at 0.4 and 1 μg/ml of BPA.

**Table 1:** Results of chromosomal structural aberrations

**Total**	**Breakages**	**Aberrant Cells**	**R**	**Tr**	**Ctg**	**Csg**	**Ctb**	**Csb**	**Fra**	**Seen Cells**	**BPA** **(µg/ml)**	**Cell Groups**
56	59	40	0	3	0	0	8	0	45	200	0	**MCF-7**
69	71	55	0	2	0	0	4	0	63	200	0.4	
88	86	80	0	0	2	0	0	0	86	200	1	
234	236	185	9	9	16	0	20	0	180	200	4	
208	197	168	3	0	14	0	28	5	158	200	40	
72	72	59	0	0	0	0	0	0	72	200	100	
0	0	0	0	0	0	0	0	0	0	200	0	**Male Amniocytes**
2	2	2	0	0	0	0	0	0	2	200	0.4	
32	32	32	0	0	0	0	0	0	32	200	1	
50	50	49	0	0	0	0	0	0	50	200	4	
50	50	48	0	0	0	0	0	0	50	200	40	
28	28	28	0	0	0	0	0	0	28	200#	100	
0	0	0	0	0	0	0	0	0	0	200	0	**Female Amniocytes**
0	0	0	0	0	0	0	0	0	0	200	0.4	
50	51	48	2	0	0	1	0	0	47	200	1	
72	70	69	0	0	0	2	0	0	70	200	4	
0	0	0	0	0	0	0	0	0	0	0*	40	
0	0	0	0	0	0	0	0	0	0	0*	100	

#: Because of high BPA cytotoxicity in this concentration, the analyzed metaphases were less than 200 which were extrapolated to 200;

*: Because of high BPA cytotoxicity in this concentrations, no analyzable metaphase was observed; Fra: fragments; DM: double minute; M: marker chromosome; Csb: chromosomal breakage; Ctb: chromatid breakage; Csg: chromosomal gap; Ctg: chromatid gap; Tr: triaradial; R: ring chromosome.

Chromosomal fragments were the most frequent structural aberrations induced by BPA in this study. The patterns of fragments and breakages induction were similar to the pattern of total structural aberrations (figures 4C, D). 

As it is obvious in figure 4, the number of abnormal cells and also structural aberrations decreased in higher doses. For example no analyzable female amniocyte metaphase was found at doses 40 and 100 μg/ml which could be due to cytotoxic effects of BPA and cell arrest at these doses.

**Chromosomal rearrangements**

The karyotypes of the chromosomal spreads in MCF-7 cells and amniocytes were normal using GTG banding technique.

**Numerical aberrations**

Although, the numerical variations in BPA exposed MCF-7 cells were completely observable, but it didn't follow any particular pattern of chromosomes number variation after exposure to different concentrations of BPA (Table 2).

**Fig. 5:**
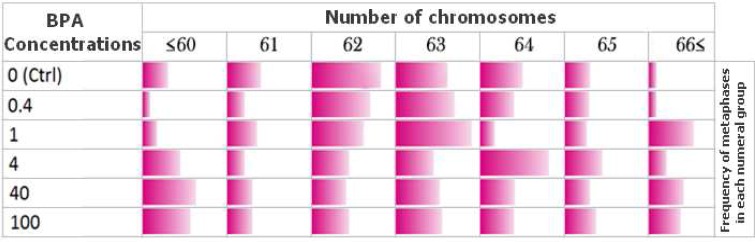
Chromosomal numerical variations in BPA exposed MCF-7 cell line. The color spectrum magnitudes indicate the relative frequency of metaphases in each chromosomal numerical group (each column

**Table 2:** Numerical aberrations in cells exposed to different concentrations of BPA

**Cell type**	**Concentrations**	**The percentage of MCF-7 metaphases observed in different chromosome number group**						
		**≤60**	**61**	**62**	**63**	**64**	**65**	**≥66**
**MCF-7**	Negative (Ctrl)	8.8	13	30.3	21.7	17.4	8.8	-
	0.4	-	5	25	25	13	8.5	-
	1	3.7	11.1	22.3	33.3	3.7	7.4	18.5
	4	15	5	15	15	30	15	5
	40	22.6	9	13.6	18.1	13.6	9	13.5
	100	20	9	15	19	13	12	12
		**The percentage of amniocyte metaphases observed in different chromosome number group**						
		**≤43**	**44**	**45**	**46**	**47**	**48**	**≥49**
**Male Amniocytes**	Negative ( Ctrl )	-	-	-	100	-	-	-
	0.4	-	-	-	100	-	-	-
	1	-	-	3	97	-	-	-
	4	-	-	-	96	4	-	-
	40	-	-	-	93	7	-	-
	100#	-	-	12	88	-	-	-
	Negative ( Ctrl )	-	-	-	100	-	-	-
**Female Amniocytes**	0.4	-	-	-	100	-	-	-
	1	-	-	8	88	4	-	-
	4	-	-	8	53*	28	8	-
	40≠	-	-	-	-	-	-	-
	100≠	-	-	-	-	-	-	-

# : Because of high BPA cytotoxicity in this concentration, the analyzed metaphases were less than 200 which were extended to 200 and percentages were calculated from 200 cells;

≠: Because of high BPA cytotoxicity in this concentrations, no analyzable metaphase was observed;

* : p< 0.05

## Discussion

Despite the availability of information on the toxicity effects of BPA on reproductive system as an endocrine disruptor, there are still many remaining questions concerning the genotoxic effects of BPA (31-35). BPA binds to ERs that could increase breast cancer risk (22). In the present study we investigated the effects of BPA on DNA damage in ER-positive MCF-7 cell line as well as ER-positive and negative amniocytes. We have used ICC in order to confirm the presence of ERs in female amniocytes and lack of its expression in male amniocytes. The results were obtained as expected and are in agreement with the findings of Chen et al. (30). 

Cytotoxicity effects of BPA have been evaluated by MTT assay in MCF-7 cells as well as female and male amniocytes. Results expectedly showed that the amniocytes with positive ER are more sensitive to BPA (3, 18, 19). Despite the presence of ER on MCF-7 cells, this cell line showed a high tolerance to BPA compared to amniocytes that could be attributed to the cancerous features of MCF-7. The MCF-7 cell line could continue its cell cycle despite the presence of chromosome aberrations induced by relatively high BPA dosage. This result confirmed our supposition that MCF-7 cell line is a good monitoring system for genotoxic evaluation of BPA as a xenoestrogen compound. 

Because of high cytotoxicity effects of BPA, the ER-positive (female) amniocytes were not able to survive at concentrations of 40 and 100 μg/ml of BPA (figure 4). As a result, the comparison of induced structural aberrations between ER-positive and negative cells were not possible at these concentrations of BPA. But, at 1 μg/ml of BPA a notable increase of structural aberrations (geno-toxicity) in both ER-positive and negative amniocytes were seen (interestingly, with no significant difference between these two cell groups) (Figure 4). This data is in agreement with the study of Pfeifer et al. (36) and suggests that BPA not only can cause structural aberrations in amniocytes through other pathway(s) in addition to ER, but ER-mediated pathway has a smaller share. In other words, increase of structural aberrations (genotoxicity) in both ER-positive and negative amniocytes at 1 μg/ml of BPA suggest that BPA exerts most of its genotoxic effects through ER-independent pathway(s). At the other hand, dramatic increase of cell arrest or death just for ER-positive amniocytes (cytotoxicity effects of BPA) at 40 and 100 μg/ml of BPA showed that the ER-mediated pathway is more important for cytotoxic effects of BPA and probably other xenoestrogens. To the best of our knowledge, this is the first report which demons-trates the contribution of ER in cytotoxic or genotoxic effects of xenoestrogens. Details of this contribution need to be further clarified.

In contrast to MCF-7, there was almost no structural aberration in amniocytes at 0.4 μg/ml BPA (Figure 4). It shows that amniocytes as normal body cells with a perfect DNA repair system can endure this concentration of BPA in in vitro situation. On the other hand, a cancerous cell, like MCF-7 with genomic instabilities and defective DNA repair system (37, 38), showed, although not significant, a notable increase (about 19%) of structural aberrations at this concentration of BPA (Table 1). This result suggests that BPA probably has more effects on cells which show genomic instability for various reasons or patients with cancer predisposition syndromes.

According to the study of Alard et al., the notable increase of structural aberrations at 1 μg/ml of BPA or more (especially in amniocytes) could be the result of impaired DNA repair system due to genotoxic effects of BPA (26). 

Chromosome aneuploidy or numerical aberra-tion effects of BPA in amniocytes and MCF-7 cell line at different concentrations of BPA was clearly observable (Table 2). As it is obvious in figure 5, compared with the control group, the frequency of counted MCF-7 metaphases became nearly equal between all chromosomal numerical aberration groups, after 48 h exposure to 100 μg/ml of BPA. It suggests that chromosome numerical variations in MCF-7 cells were completely random, without any clear variation pattern and with no dose depen-dency. After exposure to different concentrations of BPA, the number of amniocytes with normal 46 chromosomes decreased. A significant decrease has been observed at 4 µg/ml of BPA in female amnio-cytes but not in males. 

The genotoxic investigation of BPA after exposure of ER negative and positive cells, indicates that this component could be more harmful for predisposed ER-positive cells and also has more effects in numerical aberrations and cytotoxicity, rather than chromosomal structural aberrations in these cell types. Thus, depending on the entry way of BPA, the cells may have a different toxic destination.

Different amounts of BPA level have been measured in human biological fluids and tissues in developed countries. BPA has been detected in the majority of populations in these countries. In a study by Vandenberg et al., 0.1 µg BPA per gram of placenta tissue was obtained (3). Hormann et al. showed that short-term exposure to bills or receipts printed by thermal printers can be a cause of multifold increase of BPA concentration in human plasma (39). According to our unpublished data, 1 µg BPA per ml of plasma was extracted from mice after 5 µg/kg oral administration of BPA for 35 days. 

Although, the toxic levels of BPA were not reported in the human body fluid in the literature yet, but the potential risk of BPA should not be overlooked. However, the data concerning the BPA levels in different populations is not available in developing as well as underdeveloped countries. Because of low quality of plastics industry as well as lack of efficient recycling system in these countries, it is expected that in different populations of these countries, BPA levels should be higher than in the developed ones. The same applies to workers of plastics industry and other highly exposed people. On the other hand, many types of xenoestrogens exist in human living environments and the cumulative effects of these components in in vivo situation, may have the same effects as the examined toxic levels of BPA in the present in vitro investigation.
